# Molecular characteristics, clinical significance, and immune landscape of extracellular matrix remodeling-associated genes in colorectal cancer

**DOI:** 10.3389/fonc.2023.1109181

**Published:** 2023-08-09

**Authors:** Wenlong Chen, Yiwen Wang, Haitao Gu, Yi Zhang, Cong Chen, Tingting Yu, Tao Chen

**Affiliations:** ^1^ Department of Colorectal Surgery, The First Affiliated Hospital of Nanjing Medical University, Nanjing, China; ^2^ Department of General Surgery, Shanghai General Hospital, Shanghai Jiao Tong University School of Medicine, Shanghai, China; ^3^ Department of Neurology, The First Affiliated Hospital of Nanjing Medical University, Nanjing, China; ^4^ Department of Medical Genetics, School of Basic Medical Science, Jiangsu Key Laboratory of Xenotransplantation, Nanjing Medical University, Nanjing, China

**Keywords:** extracellular matrix remodeling, colorectal cancer, prognosis, tumor microenvironment, immunotherapy

## Abstract

**Background:**

Extracellular matrix (ECM) remodeling is one of the hallmark events in cancer and has been shown to be closely related to tumor immunity. Immunotherapy has evolved as an important tool to treat various cancers and improve patient prognosis. The positive response to immunotherapy relies on the unique interaction between cancer and the tumor microenvironment (TME). However, the relationship between ECM remodeling and clinical outcomes, immune cell infiltration, and immunotherapy in colorectal cancer (CRC) remains unknown.

**Methods:**

We systematically evaluated 69 ECM remodeling-associated genes (EAGs) and comprehensively identified interactions between ECM remodeling and prognosis and the immune microenvironment in CRC patients. The EAG_score was used to quantify the subtype of ECM remodeling in patients. We then assessed their value in predicting prognosis and responding to treatment in CRC.

**Results:**

After elaborating the molecular characteristics of ECM remodeling-related genes in CRC patients, a model consisting of two ECM remodeling-related genes (MEIS2, SLC2A3) was developed for predicting the prognosis of CRC patients, Receiver Operating Characteristic (ROC) and Kaplan-Meier (K-M) analysis verified its reliable predictive ability. Furthermore, we created a highly reliable nomogram to enhance the clinical feasibility of the EAG_score. Significantly differences in TME and immune function, such as macrophages and CD8^+^ T cells, were observed between high- and low-risk CRC patients. In addition, drug sensitivity is also strongly related to EAG_score.

**Conclusion:**

Overall, we developed a prognostic model associated with ECM remodeling, provided meaningful clinical implications for immunotherapy, and facilitated individualized treatment for CRC patients. Further studies are needed to reveal the underlying mechanisms of ECM remodeling in CRC.

## Introduction

CRC is one of the most common gastrointestinal tumors worldwide and the second leading cause of cancer-related deaths (1). Currently, TNM (tumor node metastasis) staging is the most commonly used clinical staging method to guide the treatment and management of CRC patients (2). Early stage I and II CRC can be cured by surgical resection, while the standard treatment for stage III CRC is surgical resection with adjuvant chemotherapy. There are several treatment options for metastatic CRC, including surgery, chemotherapy, radiotherapy, immunotherapy, and biologic-targeted therapy (3, 4). However, under the TNM staging criteria, due to the complexity of tumors, patients at the same TNM stage still show large differences in treatment outcomes and clinical prognosis. Therefore, it is important to find better classification methods for predicting prognosis and guiding treatment for CRC patients.

Extracellular matrix (ECM) is a collection secreted by cells to provide structural and biological support to surrounding cells and its major components include collagen, elastin, and polysaccharides (5, 6). The concept of ECM remodeling can be understood as a change in the physical to biochemical properties of the ECM, i.e., a change in the overall abundance, concentration, and structure of individual ECM components, thereby altering the three-dimensional spatial topology of the pericellular matrix, its biochemical and biophysical properties at the tissue level, and thus affecting the biological behavior of the cell. The basic processes of ECM remodeling include (1) ECM deposition, mediated by a variety of proteases such as Lysyl Oxidase (LOX) and matrix metalloproteinases family, alters the abundance and composition of ECM components, thereby altering the ECM biochemical and mechanical properties; (2) chemical modification at the post-translational level, which alters the biochemical characteristics and structural features of the ECM; (3) protein hydrolytic degradation processes, which would release large amounts of bioactive ECM fragments and ECM binding factors and may be required to remove cellular constraints (e.g., physical barriers to migration); and (4) physical remodeling mediated by specific proteases such as procollagen-lysine, 2-oxoglutarate 5-dioxygenase 2 (PLOD2), which affects cellular behavior by aligning ECM fibrils and stabilizing the cross-linking of ECM proteins (7, 8). More importantly, this process of matrix remodeling is accompanied by complex cell-matrix biochemical signaling and molecular communication, which has a profound influence on cell behavior. In fact, the ECM is constantly undergoing dynamic remodeling and is regulated by a variety of bioactive molecules, signaling pathways, and substances released when the ECM itself is damaged. In many solid tumors such as CRC, ECM remodeling occurs as a result of collagen crosslinking and increased stiffness, and it has been demonstrated that tumor cells can detect changes in the mechanical stress of the microenvironment in which they are exposed and thus alter cell biological behavior, such as focal adhesion assembly, changes in cell contractility, overexpression of EMT markers, and upregulation of various cellular external to internal signaling cascades such as PI3K and ERK signaling (9, 10). In addition, ECM remodeling in distant organs will create favorable conditions for tumor cell metastasis prior to the development of distant metastasis (11). Therefore, tumor progression is accompanied by dysregulation of ECM remodeling.

Although only a part of CRC patients can benefit from immunotherapy in clinical practice, its importance in the comprehensive treatment of CRC has gradually emerged (12). Immune function is dependent on the structural composition and physical properties of the ECM, and the dynamic evolution of the ECM shapes a relatively immunosuppressive environment for tumor cells, suppressing both innate and adaptive immune responses (13). The simplest explanation for this is that the increased ECM density provides a physical barrier that prevents tumor cells from interacting with immune cells during the process of ECM remodeling. In recent years, with the gradual in-depth research between ECM remodeling and tumor immunity, it has been proved that the movement and metabolism of immune cells and T cell phenotype are regulated by ECM components such as collagen, which is also directly related to the development of tumor (14). Moreover, ECM remodeling eliminates the enhancement of tumor-derived exosome diffusion and subsequent cancer-associated fibroblast (CAF) induction (15). These results suggest that the mechanism of ECM remodeling and tumor immunity is far more complex than imagined.

In this study, we grouped patients using ECM remodeling-related genes and established a prognostic model based on MEIS2 and SLC2A3. Besides, a nomogram that accurately predicted patient survival was constructed. Further, we assessed the differences between high- and low-risk groups in clinical characteristics, molecular features, immune function, and drug sensitivity. In conclusion, our work constructed a valid prognostic model and provides new insights into the immunotherapy of CRC patients.

## Materials and methods

### Data collection

The process of this work is shown in **Figure S1**. In this study, GSE39582 (16) and GSE17536 (17) were selected as training cohort (n = 758) from the Gene Expression Omnibus (GEO) database, 618 CRC samples from The Cancer Genome Atlas (TCGA) were applied as testing cohort. After excluding patients who lacked important clinical information such as overall survival (OS) and AJCC stage, we conducted data normalization to avoid batch effects. The gene set containing 69 genes related to ECM remodeling was obtained from the AmiGO database with the keyword “ECM remodeling” and the restriction “Homo sapience” (18) (**Table S1**).

### Consensus clustering analysis

Cluster analysis was performed using the “ConsensusClusterPlus” package, using agglomerative km clustering with a 1-Pearson correlation distances and resampling 80% of the samples for 10 repetitions. The optimal number of clusters was determined using the empirical cumulative distribution function plot (19).

### Association between molecular patterns with the clinical characteristics and prognosis of CRC

We integrated the patients’ clinical information such as survival time, survival status, age, gender, grade, and AJCC stage. Kaplan-Meier analyses obtained by the “Survival” and “SurvMiner” packages were used to assess OS differences between different groups (20).

### Relationship of molecular patterns with TME in CRC

We used the ESTIMATE algorithm to assess the Stromalscore, Immunescore, and TMEscore of CRC patients. The CIBERSORT algorithm and MCPcounter algorithm were used to calculate the level of immune cell and stromal cell infiltration in each patient. We evaluated the association between risk scores, expression levels of genes involved in model construction, and immune cell infiltration, respectively. Then we compared subgroup differences in immune checkpoint expression and immune function. The “GSVA” package was used for GSVA analysis. We downloaded the subset c2.cp.kegg.v7.4.symbols.gmt to evaluate the relevant pathways and molecular mechanisms, set the minimum gene set to 5, the maximum gene set to 5000, and calculated the enrichment score of each sample in each gene set. Finally, the enrichment score matrix is obtained for further analysis.

### Identification of DEGs and functional enrichment analysis

We obtained 195 differentially expressed genes (DEGs) between ECM remodeling subgroups using the “Limma” package under | logFC | ≥ 1 and p < 0.05. Then “clusterProfiler” package was used to perform Gene Ontology (GO) function and the Kyoto Encyclopedia of Genes and Genomes (KEGG) pathway enrichment analysis based on these DEGs (21).

### Development of the ECM remodeling-associated prognostic EAG_Score

We constructed an EAG_Score to quantitatively assess the degree of ECM remodeling in each patient. Based on 195 DEGs, we screened 130 DEGs associated with prognosis using univariate Cox regression (uniCox) analysis and subsequently performed regression analysis using the “glmnet” package. LASSO-cox analysis was used to construct a prognostic model. the EAG_Score was calculated as: EAG_Score = ∑ (gene Expression ∗ gene coefficient). Based on the median EAG_Score, we divided the patients into high and low-risk groups and performed PCA analysis using the “stats” package (version 3.6.0). Specifically, we first performed z-score transformation of the patients’ gene expression profiles and further performed dimensionality reduction analysis using the prcomp function to obtain the reduced matrix.

### Clinical significance analysis of the prognostic EAG_Score

After excluding patients with missing data, we integrated patients’ clinical information and EAG_Score for uniCox and multivariate Cox regression (multiCox) analysis; we performed ROC analysis using the pROC package (version 1.17.0.1) to obtain AUC (Area Under Curve) values. Specifically, AUC values and Confidence Intervals (CI) were evaluated using CI function of the package to obtain the final AUC results. We analyzed whether the EAG_Score model could be used as an independent prognostic predictor. In addition, we analyzed the differences in EAG_Score between the subgroups obtained by different clustering analysis methods.

### Establishment of a predictive nomogram

Combined with the patients’ EAG_Score and other clinicopathological features, the “rms” package was used to plot a nomogram. We conducted ROC analysis to explore the prognostic predictive power of these clinical features, particularly in predicting the patients’ 1-year, 3-year, and 5-year OS. Calibration curves were used to validate the predictive accuracy of the column line graphs.

### Immunohistochemical analysis

By searching the Human Protein Atlas (HPA) database, we obtained immunohistochemical staining images to determine protein expression of MEIS2 and SLC2A3 in normal and CRC tissues. HPA003256 and CAB002763 are antibodies to MEIS2 and SLC2A3, respectively.

### Drug sensitivity analysis

Setting a filter condition of p < 0.001, we predicted patients’ IC50 values for drugs commonly used in clinical practice using the “pRRophetic” package and compared them in high and low-risk groups (22).

## Results

### ECM remodeling related genes in CRC

**Figure 1:**
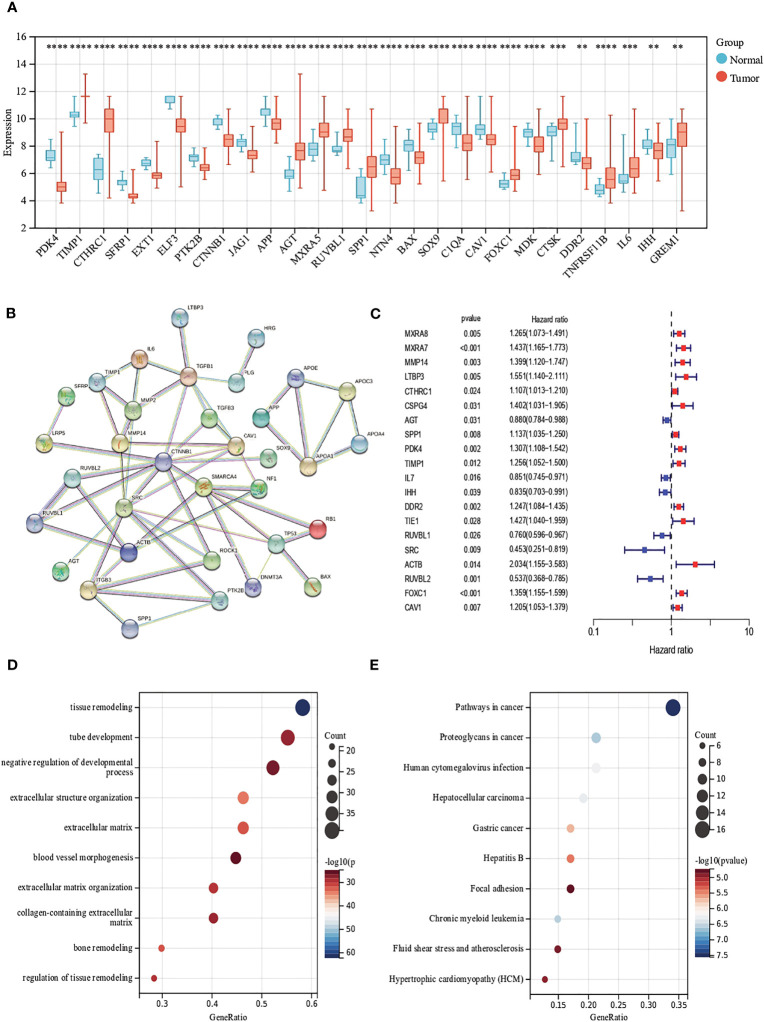
Evaluation of ECM remodeling associated genes in CRC. **(A)** Expression distributions of ECM remodeling associated genes between CRC and normal tissues. **(B)** The PPI network acquired from the STRING database among the ECM remodeling associated genes. **(C)** Forest map of ECM remodeling associated genes with prognostic significance (p<0.05). **(D, E)** GO and KEGG enrichment analysis of ECM remodeling associated genes. (p<0.01 **; p<0.001 ***; p<0.0001 ****).

First, we analyzed the mRNA expression data in the training cohort, after eliminating the batch effect (**Figure S2**), we identified the expression levels of 69 ECM remodeling-associated genes in tumor samples and normal samples and it was found that most of the genes were down-regulated in tumor tissue (**Figure 1A**). PPI networks were mapped using an online tool (https://cn.string-db.org) to explore the association between ECM remodeling genes. The results showed that CTNNB1, SMARCA4, MMP14, SRC, and ACTB were hub genes (confidence score = 0.900) (**Figure 1B**). Subsequently, we explored the prognostic value of these genes by uniCox analysis. 20 genes, including MXRA8, MXRA7, and MMP14, have been shown to be associated with the prognosis of patients (**Table S3**; **Figure 1C**). GO and KEGG analysis showed that these genes were mainly related to the regulation of tissue and ECM remodeling, cancer pathways such as liver cancer and gastric cancer (**Figures 1D, E**).

### Generation of ECM remodeling subgroups in CRC

**Figure 2:**
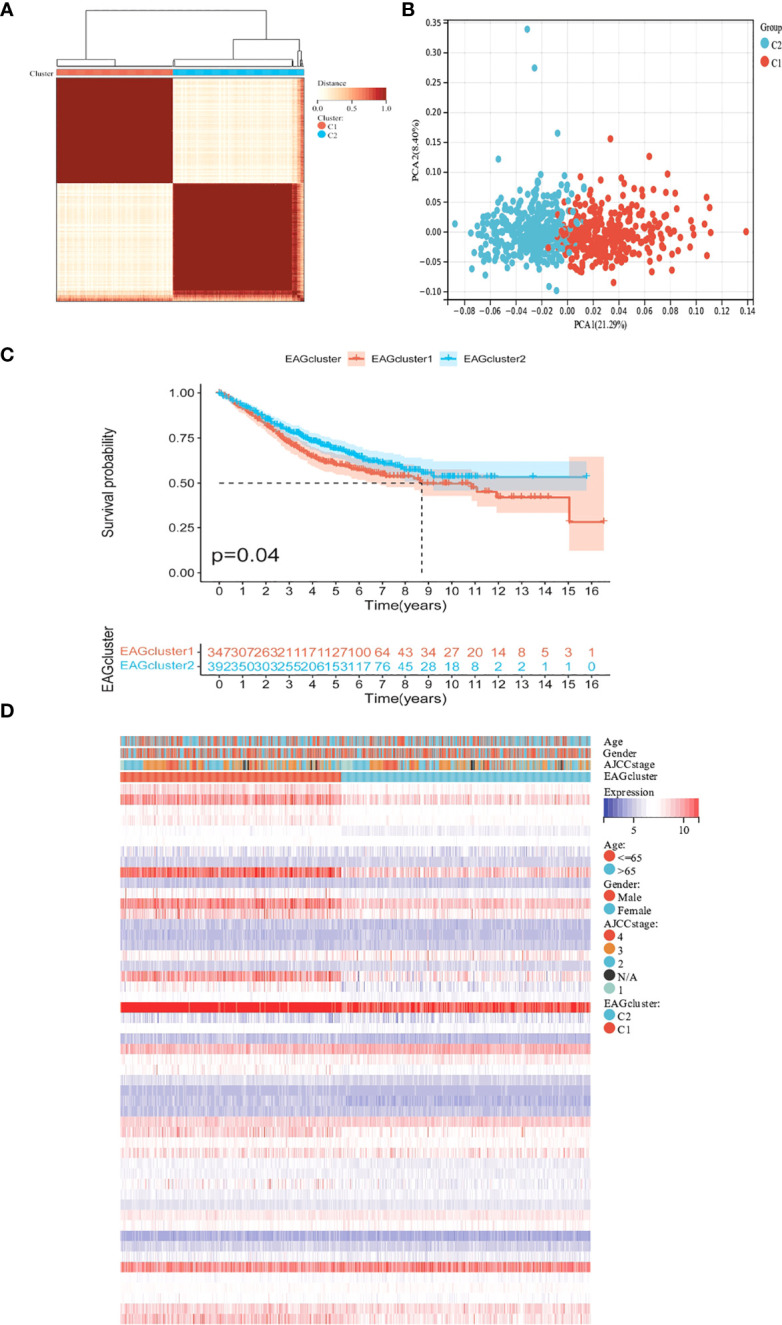
Classification of ECM remodeling associated genes subgroups and clinicopathological and biological characteristics of two distinct subtypes. **(A)** Consensus analysis matrix heatmap defining two clusters (k = 2) and their correlation area. **(B)** PCA analysis showed significant differences in the transcriptome between the two subgroups. **(C)** Kaplan–Meier curves for OS of the EAGclusters. **(D)** Differences in clinicopathologic characteristics and expression levels of ECM remodeling associated genes between EAGcluster1 and EAGcluster2.

To further determine the relationship between ECM remodeling and CRC, we performed the clustering analysis based on ECM remodeling-associated genes and the result showed that the best clustering variable was 2. The patients were classified into EAGcluster 1 (n = 347) and EAGcluster 2 (n = 392) (**Figure 2A**). PCA analysis showed the reliability of the grouping (**Figure 2B**). We compared the OS between the groups and observed a significant survival difference (**Figure 2C**). In addition, as shown in **Figure 2D**, EAGcluster 1 possessed higher levels of ECM remodeling-related gene expression and was associated with a more advanced AJCC stage.

### Characteristics of the TME in different subgroups

**Figure 3:**
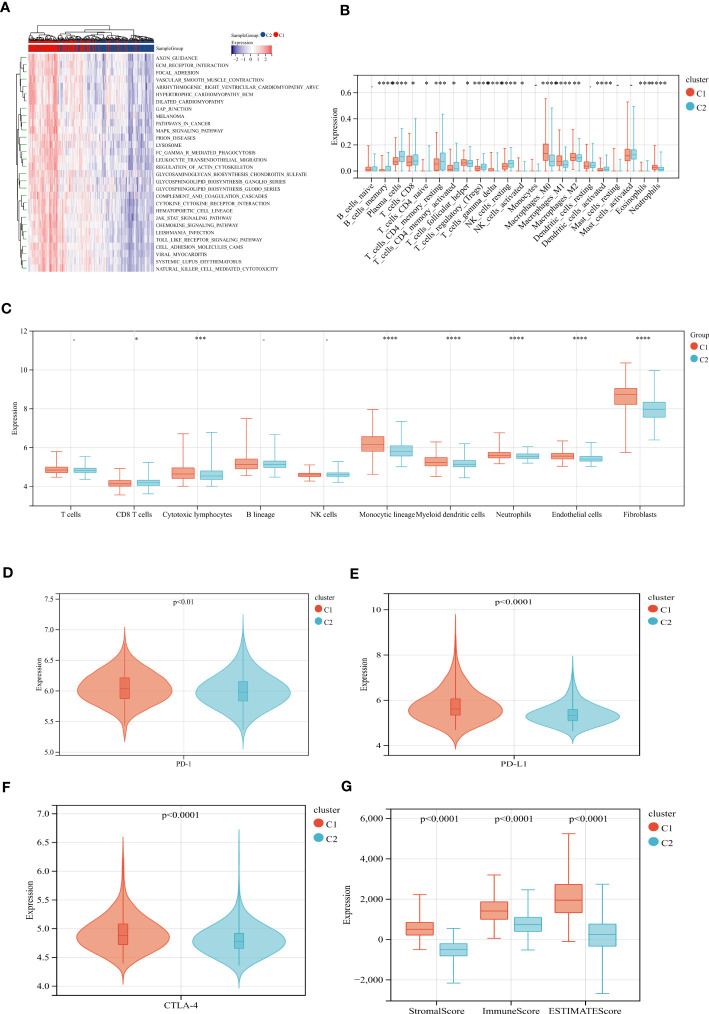
Differences in tumor immune microenvironment between EAGcluster1 and EAGcluster2. **(A)** GSVA of biological pathways between two distinct subgroups. **(B)** Abundance of 22 infiltrating immune cell types in the two subgroups. **(C)** Abundance of 8 infiltrating immune cell types and 2 stromal cell types in the two subgroups. **(D–F)** Expression levels of PD-1, PD-L1, and CTLA-4 in the two subgroups. **(G)** Correlations between the two subgroups and TME score. (p<0.05 *; p<0.001 ***; p<0.0001 ****; p>0.05) "-" symbol means p>0.05.

We performed GSVA analysis to compare the differences in ECM remodeling-related enrichment pathways between these subgroups, the result showed that ECM signaling exchanges (such as ECM receptor interaction, glycosaminoglycan biosynthesis), focal adhesion and multiple signaling pathways (JAK-STAT signaling pathway, MAPK signaling pathway) were enriched in EAGcluster1 (**Figure 3A**). The Cibersort algorithm was used to calculate the infiltration of 22 immune cells for each tumor sample (**Figure 3B**) and we observed significant differences between the two subgroups, T_cells_follicular_helper, Macrophages, Neutrophils were found to be relatively higher in EAGcluster 1, while T_cells_CD8, T_cells_CD4_memory_resting, T_cells_CD4_memory_activated, Tregs, and NK_cells_resting were higher in EAGcluster 2, the results of MCPcounter analysis showed that EAGcluster1 had a lower abundance of cytotoxic lymphocytes and a higher abundance of fibroblasts and endothelial cell infiltration than EAGcluster 2 (**Figure 3C**). Then We compared several key immune checkpoints (PD-1, PD-L1, CTLA-4) and observed that EAGcluster 1 has a higher expression level compared to EAGcluster2 (**Figures 3D–F**). Furthermore, to better understand the link between ECM remodeling and tumor immunity, TME scores (including StromalScore, ImmuneScore, and ESTIMATEScore) were calculated using the ESTIMATE algorithm, and the results showed that the EAGcluster 1 had a higher TME score (**Figure 3G**).

### Identification of gene subgroups based on DEGs

**Figure 4:**
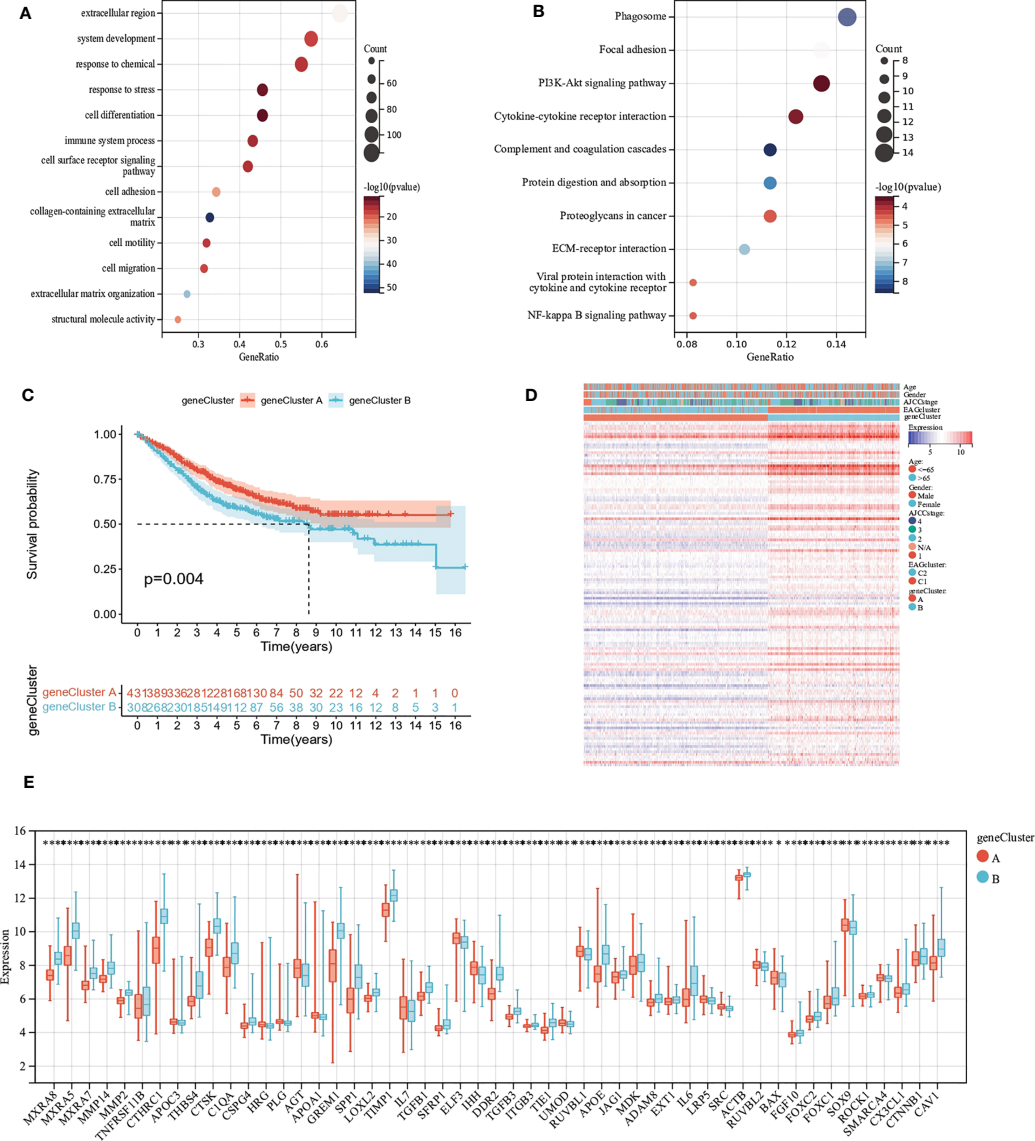
Identification of gene subgroups based on DEGs. **(A, B)** GO and KEGG enrichment analysis of DEGs. **(C)** Kaplan–Meier curves for OS of the two gene clusters. **(D)** Differences in clinicopathologic characteristics and expression levels of ECM remodeling associated genes between the two gene clusters. **(E)** Differences in the expression of ECM remodeling associated genes among the two gene clusters. (p<0.05 *; p<0.01 **; p<0.001 ***; p<0.0001 ****).

To explore the potential biological activity of ECM remodeling subgroups, we obtained 195 DEGs related to ECM remodeling using the “limma” package and performed functional enrichment analysis (**Tables S6, S7**). GO analysis showed that DEGs were mainly enriched in biological processes such as cell adhesion, cell differentiation, and cell motility (**Figure 4A**). KEGG analysis showed significant enrichment in the PI3K-Akt signaling pathway, Phagosome, Cytokine-cytokine receptor interaction, etc (**Figure 4B**). Subsequently, we discussed the prognostic value of DEGs using uniCox analysis and obtained a total of 28 prognosis-related genes at p < 0.001 (**Table S8**). We divided patients into two geneClusters based on the expression of prognostic genes. We found that patients in geneCluster A had better OS compared to geneCluster B (**Figure 4C**), and geneCluster B had more abundant DEGs expression and was associated with later AJCC stage (**Figure 4D**). In addition, as expected, there was significant differential expression of ECM remodeling-related genes between geneClusters (**Figure 4E**).

### Development and validation of the prognostic AAG_Score

**Figure 5:**
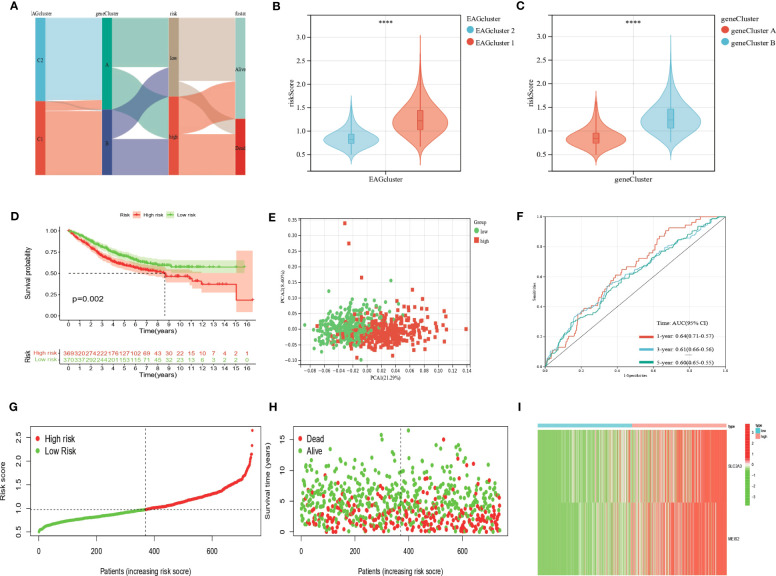
Construction of the EAG_score in the entire cohort. **(A)** Alluvial diagram of subgroup distributions in groups with different EAG_scores and clinical outcomes. **(B)** Differences in EAG_score between the two EAG clusters. **(C)** Differences in EAG_score between the two gene clusters. **(D)** Kaplan–Meier analysis of the OS between high- and low-risk group. **(E)** PCA analysis based on the prognostic signature. **(F)** ROC curves to predict the sensitivity and specificity of 1-, 3-, and 5-year survival according to the EAG_score. **(G, H)** Ranked dot and scatter plots showing the EAG_score distribution and patient survival status. **(I)** Expression patterns of 2 selected prognostic genes in high- and low-risk groups. (p<0.0001 ****).

We developed the EAG_Score to assess the prognostic predictive ability of DEGs in CRC patients. LASSO and multiCox analysis for 130 prognosis-related DEGs were conducted to establish an optimal predictive model. Finally, we obtained two genes (MEIS2 and SLC2A3) and the formula of EAG_score: risk score = (0.167125186316921 * expression of SLC2A3) + (0.144964442480063 * expression of MEIS2). **Figure 5A** displayed the patients’ distribution in the different groups. Besides, we found EAGcluster 1 and geneCluster A had higher risk score (**Figures 5B, C**). Subsequently, patients were divided into high-risk and low-risk groups according to the median EAG_Score, Kaplan-Meier analysis indicated that low-risk patients had a better OS over high-risk patients (**Figure 5D**). PCA analysis showed a good distribution of patients in high and low-risk groups (**Figure 5E**). The AUC values of the model predicting patients’ OS at 1, 3, and 5 years were 0.64 (95% CI = 0.71-0.57), 0.61 (95% CI = 0.66-0.56), and 0.60 (95% CI = 0.65-0.55), respectively (**Figure 5F**). Compared with other existing prognostic models for CRC, such as the ferroptosis-related genes prognostic model (AUC = 0.64, 0.64, 0.71 for 1,3,5-year OS, respectively) (23), the platelet-related prognostic model (AUC = 0.722, 0.706, 0.689 for 1,3,5-year OS,respectively) (24), and the anoikis and immune-related genes prognostic model (AUC = 0.671, 0.634, 0.638 for 1,3,5-year OS,respectively) (25), our model also demonstrated good predictive performance. With the increase of EAG_Score, the OS of patients decreased and the mortality rate gradually increased (**Figures 5G, H**). **Figure 5I** shows the expression heatmap of MEIS2 and SLC2A3.

**Figure 6:**
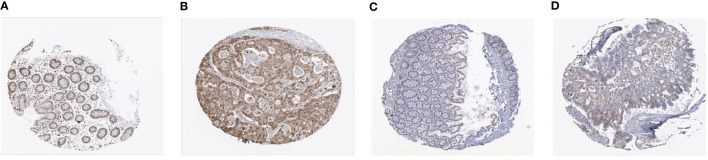
Representative immunohistochemistry images of MEIS2 and SLC2A3 in CRC tissues and normal tissues. **(A, B)** MEIS2 in normal tissues and CRC tissues. **(C, D)** SLC2A3 in normal tissues and CRC tissues.

In addition, our prognostic model also showed good predictive power in validation cohort (**Figure S3**). The expressions of MEIS2 and SLC2A3 were verified by The Human Protein Atlas (**Figure 6**).

## Clinical correlation analysis of the prognostic EAG_Score

**Figure 7:**
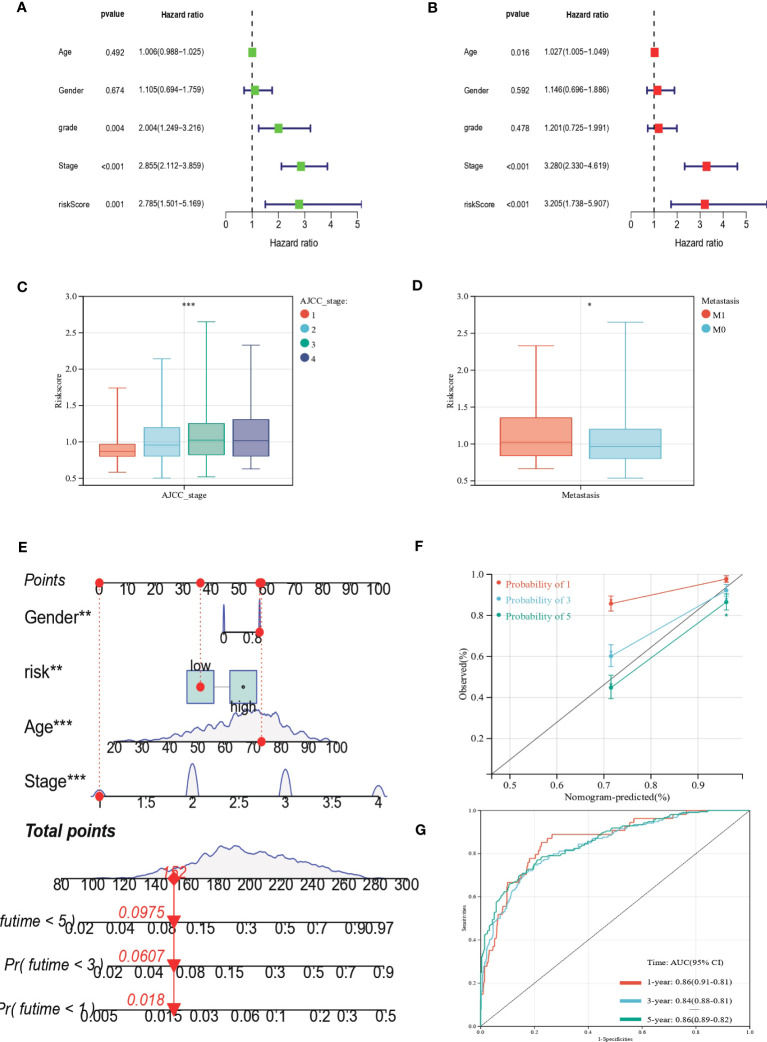
Clinical correlation analysis of the prognostic EAG_Score and establishment of the prognostic nomogram. **(A, B)** uniCox and multiCox analysis showed the prognostic value of the EAG_score. **(C)** Correlation between risk score and AJCC_stage. **(D)** Correlation between risk score and CRC metastasis. **(E)** Nomogram for predicting the 1-, 3-, and 5-year OS of CRC patients in the entire cohort. **(F)** Calibration curve of the prognostic nomogram. **(G)** ROC curves of the prognostic nomogram for 1-, 3-, and 5-year OS in CRC. (p<0.05 *; p<0.01 **; p<0.001 ***).

We performed uniCox and multiCox analyses to determine the independent prognostic value of EAG_Score (**Figures 7A, B**). The forest plot showed that EAG_Score could be used as an independent factor to predict the prognosis of patients. In addition, to explore the relationship between EAG_Score and clinical characteristics, we discussed the correlation between clinical information such as age, gender, and AJCC stage, the results showed that later AJCC stage was associated with a higher risk score (**Figure 7C**). Moreover, patients with metastasis had a higher EAG_Score (**Figure 7D**). Overall, a higher risk score means a higher risk of metastasis and a worse prognosis.

### Construction of a nomogram to predict patients’ prognosis

Given the good predictive efficacy of the model, we further developed a nomogram that could predict the OS of patients at 1, 3, and 5 years based on the clinical characteristics (**Figure 7E**). The calibration curve showed that the nomogram had a great accuracy between actual observations and predicted values (**Figure 7F**). In the ROC curve, the AUC values of the nomogram for predicting the 1-year, 3-year, and 5-year OS of patients were 0.86 (95%CI = 0.91-0.81), 0.84 (95%CI = 0.88-0.81) and 0.86 (95%CI = 0.89-0.82), respectively (**Figure 7G**).

### Assessment of TME, checkpoints, and immune function in distinct groups

**Figure 8:**
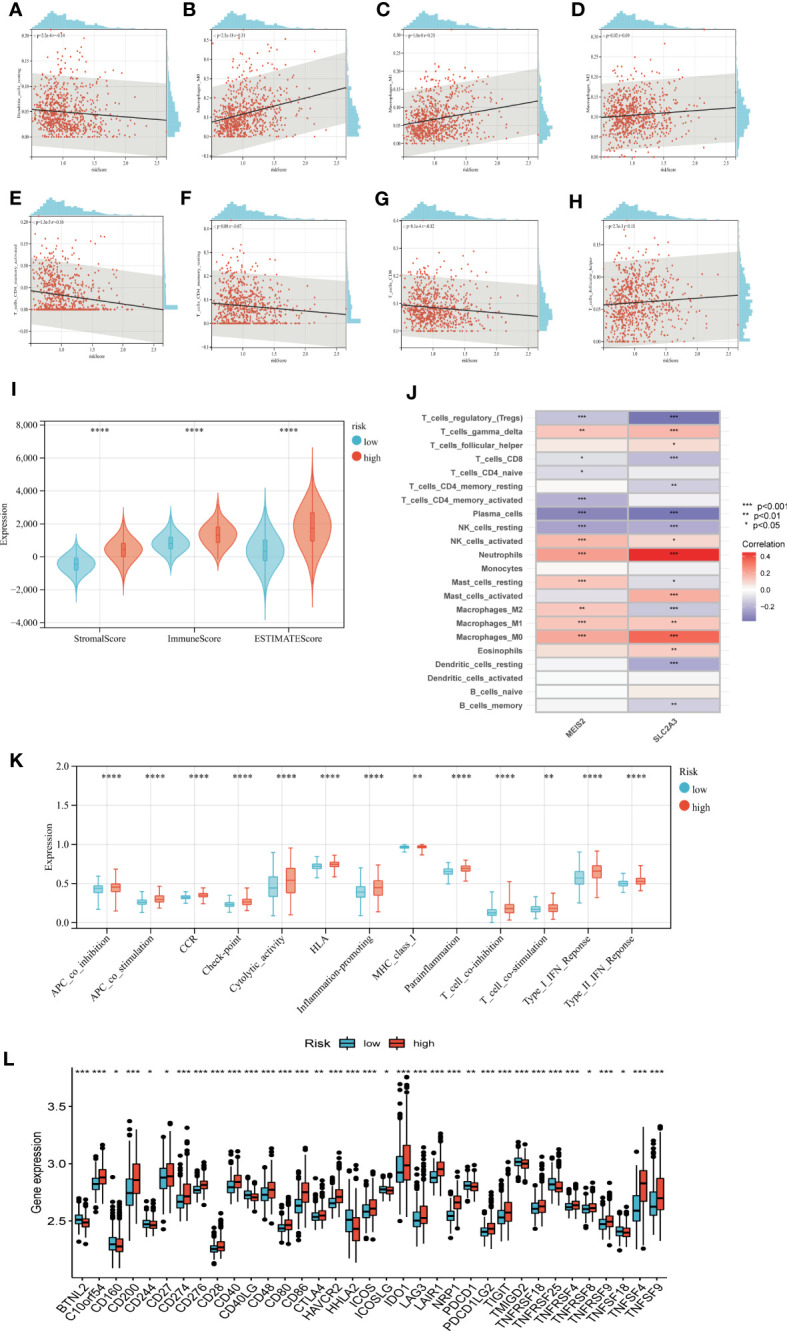
Evaluation of TME, checkpoints and immune functions between the two groups. **(A–H)** Correlations between EAG_score and immune cell types. **(I)** Correlations between EAG_score and both immune and stromal scores. **(J)** Correlations between the abundance of immune cells and genes involved in development of the prognostic model. **(K)** Assessment of differences in immune function between the two groups **(L)** Expression of 35 common immune checkpoints in the two groups. (p<0.05 *; p<0.01 **; p<0.001 ***).

We calculated the correlation between immune cell abundance and EAG_Score. As shown in (**Figures 8A–H**), EAG_Score was positively correlated with Macrophages_M0, Macrophages_M1, Macrophages_M2, T_cells_follicular_helper, and negatively correlated with Dendritic_cells_resting, T_cells_CD4_memory_activated, T_cells_CD4_memory_resting, T_cells_CD8. In addition, EAG_Score was associated with higher StromalScore, ImmuneScore, and ESTIMATEScore (**Figure 8I**). We found a significant correlation between the genes involved in the model construction and most immune cells’ infiltration levels (**Figure 8J**). **Figure 8K** demonstrated that certain immune functions including APC_co_stimulation, CCR, HLA, and T_cell_co-stimulation, differed significantly between the two distinct groups. Furthermore, we compared 35 common immune checkpoint inhibitors between high- and low-risk groups, such as PD-1, PD-L1, CTLA-4, lymphocyte-activation gene 3, and tumor necrosis factor superfamily, and they were discrepantly represented in the two risk subgroups (**Figure 8L**).

### Drug sensitivity analysis

**Figure 9:**
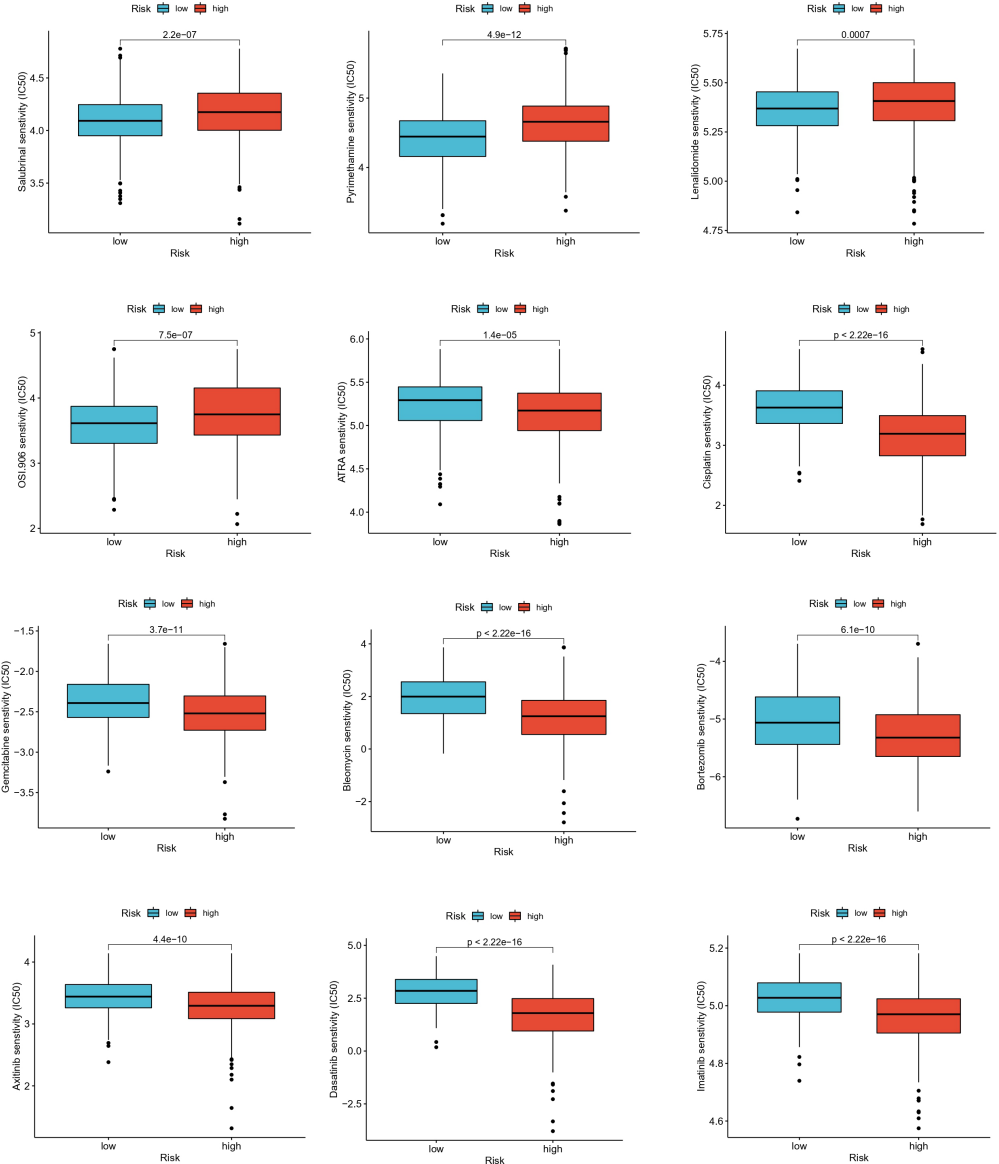
Relationships between EAG_score and drug sensitivity.

To assess the ability of EAG_Score in predicting clinical drug therapy sensitivity in CRC patients, we calculated the IC50 values for each patient for 138 drugs using the “pRRophetic” package. We found that patients with low EAG_Score may have positive responses to Salubrinal, Pyrimethamine, Lenalidomide, and OSI-906, while patients with high EAG_Score may have positive responses to ATRA, Cisplatin, Gemcitabine, Bleomycin Bortezomib, Docetaxel, Doxorubicin, Etoposide and some targeted drugs such as Axitinib, Dasatinib, Imatinib, Sunitinib, Nilotinib, etc (**Figure 9**). In conclusion, these results suggested that ECM remodeling genes were correlated with drug sensitivity.

## Discussion

In cancer, the uncontrolled growth of cells remodels the ECM, this regulates cell-cell and cell-ECM interactions in turn and has profound effects on the biological behavior of cells (26). Various ECM components have pro- or anti-apoptotic effects, and dysregulation of the homeostasis of the ECM microenvironment is associated with tumor apoptotic evasion and progression (27). Clinically speaking, increased tissue stiffness can be observed in many solid tumors such as CRC, mainly due to alterations in tumor tissue fibrosis caused by increased synthesis and cross-linking of collagen (28). Tumor cells, CAF, and immune cells play a decisive role in this process, while this change in tissue remodeling can in turn induce CAF, stromal cells to secrete various cytokines, growth factors, chemokines, and exosomes, this process provides a prerequisite for tumor cell proliferation and metastasis (29). Besides, a growing number of studies have demonstrated that there is an inextricable relationship between ECM remodeling and tumor immunity (30, 31). CDH11 promotes immunosuppression and ECM deposition to support the growth of pancreatic tumors and resistance to gemcitabine (32). Versican (VCAN), a large matrix proteoglycan with immunoregulatory activity, has been reported to prevent the interaction between hyaluronan and T cells to inhibit adhesion and migration (33), and versican-derived matrikines regulate Batf3-dendritic cell differentiation and promote CD8^+^ T-cell infiltration in CRC (34). In addition, Pirfenidone, an anti-fibrotic drug, promotes immune infiltration and enhances the efficacy of PD-L1 blockers in mouse models (35). Therefore, it is of great clinical importance to further investigate the association between ECM remodeling and tumor development and immunotherapy.

In the current research, using data from GEO dataset, we evaluated the expression of genes associated with ECM remodeling in CRC and normal tissues, and patients could be classified into two different subtypes based on ECM-related genes and DEGs, respectively. Moreover, we observed a significant difference in clinical features and prognosis, immune cell and stromal cell infiltration between the different subtypes. We developed the EAG_Score model and found that EAGcluster1 and geneClusterA had higher EAG_Score and worse prognosis, which confirmed the role of ECM remodeling in CRC prognosis. In addition, the EAG_Score was identified as an independent predictor of prognosis for CRC patients, and the ROC curve validated its reliable predictive ability for 1-year, 3-year, and 5-year OS. Furthermore, the ECM remodeling-related prognostic nomograms showed good agreement between predicted and actual survival outcomes and a better prognostic ability than the TNM stage. In general, increases in infiltrating CD8^+^ T cells have been associated with longer OS (36), and consistent with that, we found the high-risk group showed more pronounced immunosuppressive features: with higher levels of Macrophages_M0, Macrophages_M1, Macrophages_M2 and lower levels of T_cells_CD4_memory_activated, T_cells_CD8, etc. Besides, the high-risk group had more abundant expression of ECM remodeling-related genes, and higher stromal and immune scores, suggesting that there is a relationship between ECM remodeling and tumor immunity that cannot be ignored. Considering the differences in immune function and immune checkpoint expression, we speculated that the high-risk group may have a higher sensitivity to immunotherapy. As research on the connection between the ECM and tumor immunity continues, ECM remodeling, structural plasticity, and mechanical forces are increasingly recognized as key factors in immune cell migration and spatial distribution, activation, and immune synapse formation (37–39). Therefore, targeting the ECM-mediated immunosuppressive microenvironment in combination with other systemic treatment strategies for CRC may enhance the efficacy of these therapies. Notably, capecitabine has been shown to inhibit the expression of CTLA-4 in CRC cells, which may combine immunotherapy with chemotherapy in the comprehensive treatment of CRC (40). These findings show promising prospects for targeting immune checkpoints in the integrated treatment of CRC. In recent years, although research on ECM such as targeting angiogenesis and tumor immunity has made tremendous progress (41), few clinical translations have been achieved (42), which may be partly explained by the non-specificity of drugs and complex tumor immunity (43). A systematic understanding of ECM remodeling and the complicated TME generated by stromal elements will help to identify investigational targets for the development of novel immune biomarkers and combination immunotherapy.

Meis homeobox 2 (MEIS2) belongs to TALE (three amino-acid loop extension) superfamily and is mainly involved in the Hox activity regulation by binding directly with posterior Hox proteins or indirectly with Pbx to form a homeoprotein-DNA complex, plays a crucial role in the pathogenesis of human cancer (44, 45). MEIS2 is essential for neuroblastoma cell survival and up-regulation of MEIS2 is required for the growth of AML1-ETO-positive AML (46, 47). In addition, it has been reported that MEIS2 may be associated with certain metastatic diseases (48, 49). Consistent with this, we found that mCRC patients had higher MEIS2 expression levels in our study. However, it has been also shown that high expression of MEIS2 is associated with improved prognosis of ovarian cancer (50). Therefore, further studies are needed to clarify the specific role of MEIS2 in cancer.

SLC2A3, a member of the solute carrier 2A family, encodes glucose transporter protein (51). SLC2A3 is up-regulated in a variety of tumors, such as CRC and breast cancer, and is involved in tumor progression and poor prognosis (52, 53). In cancer cells, the elevated expression of SLC2A3 helps to meet the increased glycolysis requirements and promotes the Warburg effect (54). In CRC, activation of the SLC2A3-YAP signaling pathway is a master activator that reprograms tumor metabolism and thus promotes tumor metastasis (55). Consistent with our findings, studies have indicated changes in the ECM of SLC2A3 overexpressed cells, including upregulation of osteopontin, which has been shown to mediate cell adhesion and promote tumor metastasis (56). In addition, we found a correlation between SLC2A3 and a variety of immune cells, such as CD4^+^ and CD8^+^T cells, macrophages, etc. Studies have suggested that SLC2A3 promotes the growth and drug resistance of gastric cancer cells by increasing the infiltration of M2 macrophages (57). Further exploration of the interaction between SLC2A3 up-regulated cancer cells and immune cells is of major importance and may provide new insights for cancer immunotherapy.

## Conclusion

In short, this study provides a comprehensive description of ECM remodeling and TME, prognosis, and clinical characteristics of CRC patients, reveals the important clinical significance of ECM remodeling related genes, and provides valuable insights for individualized therapy of CRC patients. However, this study has some shortcomings. First, data related to patients receiving immunotherapy are missing in our study, external validation based on prospective and large-scale clinical trials is needed to assess the predictive power of the model in the future. Second, the interaction between the genes involved in constructing the model and immune cells in CRC also needs to be tested experimentally. Third, potential mechanisms between ECM remodeling and tumor immunity should be revealed in the future.

## Data availability statement

The original contributions presented in the study are included in the article/**Supplementary Material**. Further inquiries can be directed to the corresponding authors.

## Author contributions

Conceptualization, TC and YZ. Methodology, TC and WC. Software, WC. Validation, WC, YW, CC and HG. Formal analysis, WC. Investigation, YW. Data curation, WC, HG and YW. Writing—original draft preparation, WC and ZY. Writing—review and editing, TC, CC, TY and YZ. Visualization, WC. Supervision, YZ and TY. Project administration, TC. Funding acquisition, TC, and YZ. All authors have read and agreed to the published version of the manuscript. All authors contributed to manuscript revision, read, and approved the submitted version.
