# Supplementary material for: Molecular characteristics, clinical significance, and immune landscape of extracellular matrix remodeling-associated genes in colorectal cancer
Source: Front Oncol. 2023 Aug 9;13:1109181. doi: 10.3389/fonc.2023.1109181 (PMC10446763; doi:10.3389/fonc.2023.1109181)
Supplement: Supplementary file 1 [file DataSheet_1.docx]

**Molecular Characteristics, Clinical Significance, and Immune Landscape of Extracellular Matrix Remodeling-Associated Genes in Colorectal Cancer**

**Wenlong Chen^1#^, Yiwen Wang^1#^, Yi Zhang^1^*, Tao Chen^1^***

***Correspondence:**

Tao Chen, e-mail: [taochen1381@njmu.edu.cn](mailto:taochen1381@njmu.edu.cn)

Yi Zhang, e-mail: [yizhang311@njmu.edu.cn](mailto:yizhang311@njmu.edu.cn)

**Supplementary Figures**


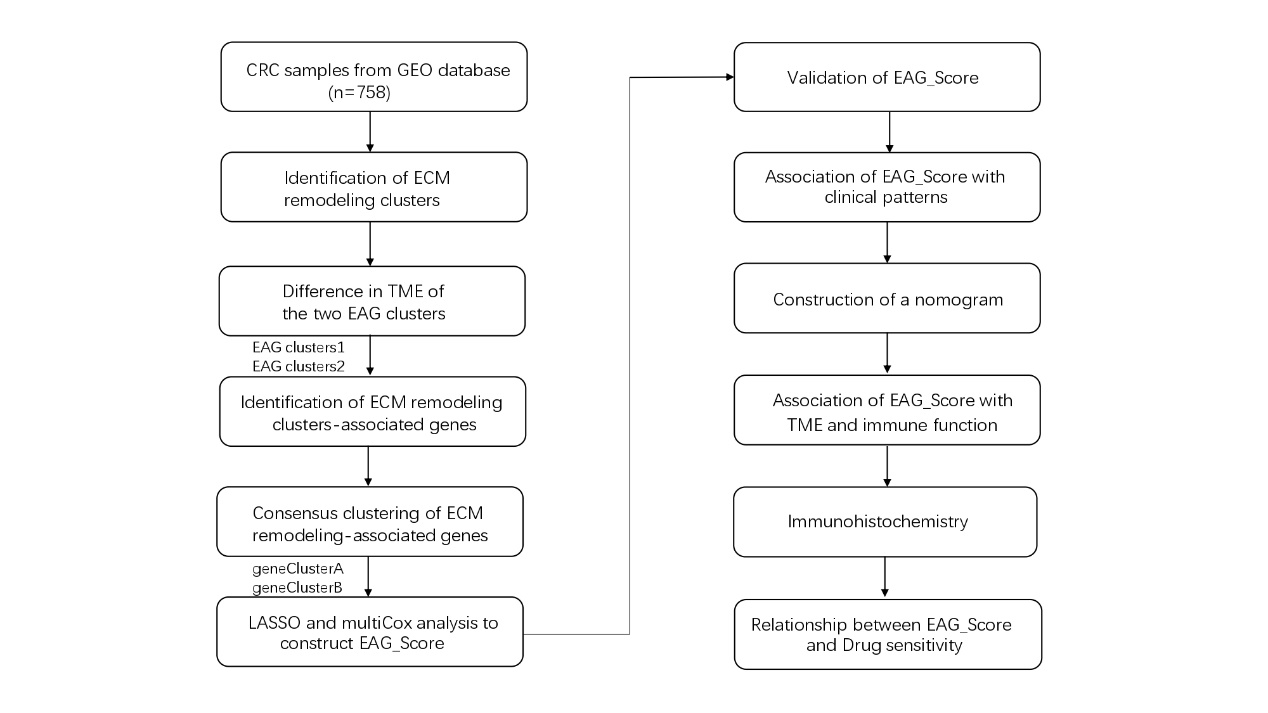


Figure S1 | The whole process of the study.


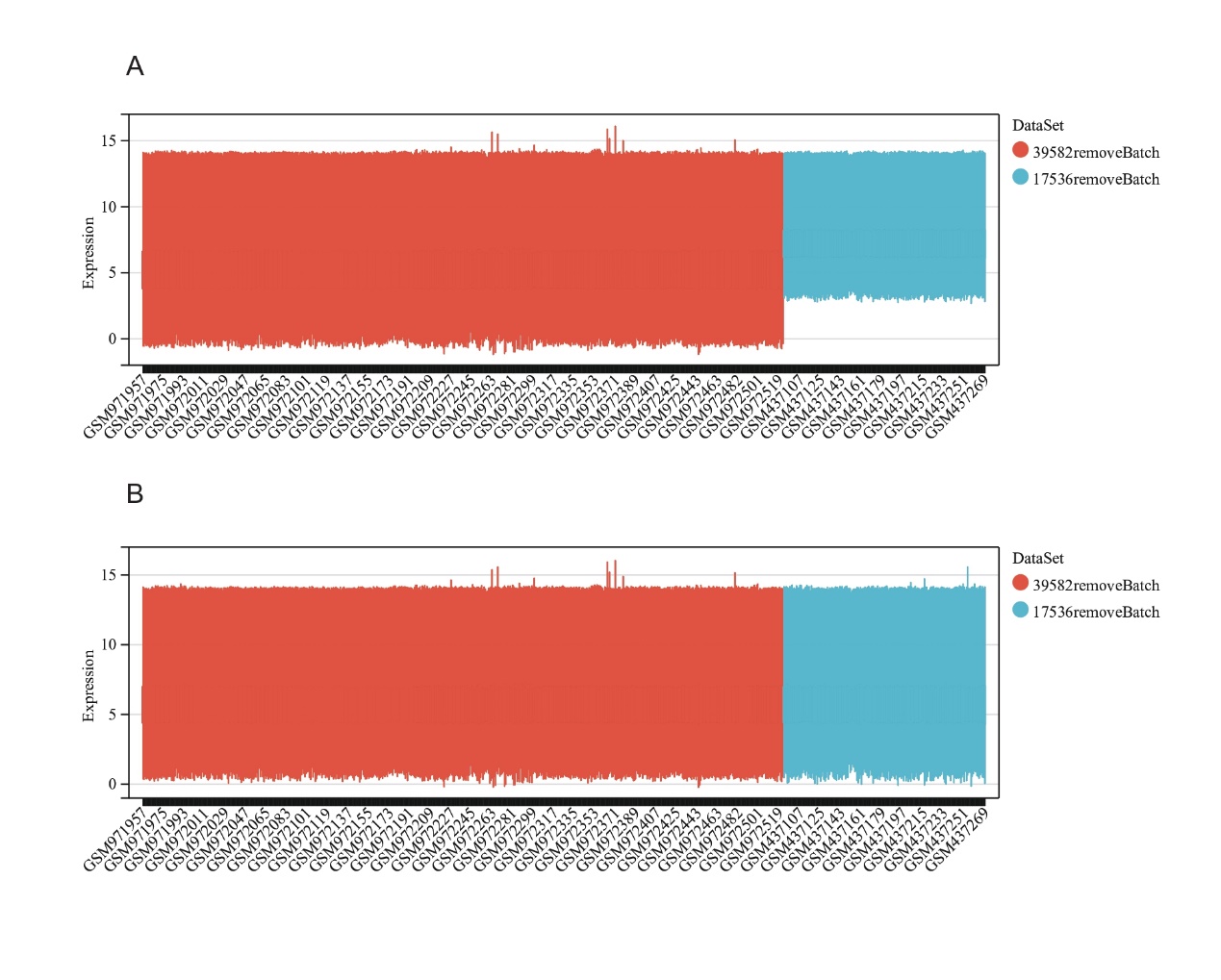


Figure S2 | Removal of batch effect. (A): Before. (B): After.


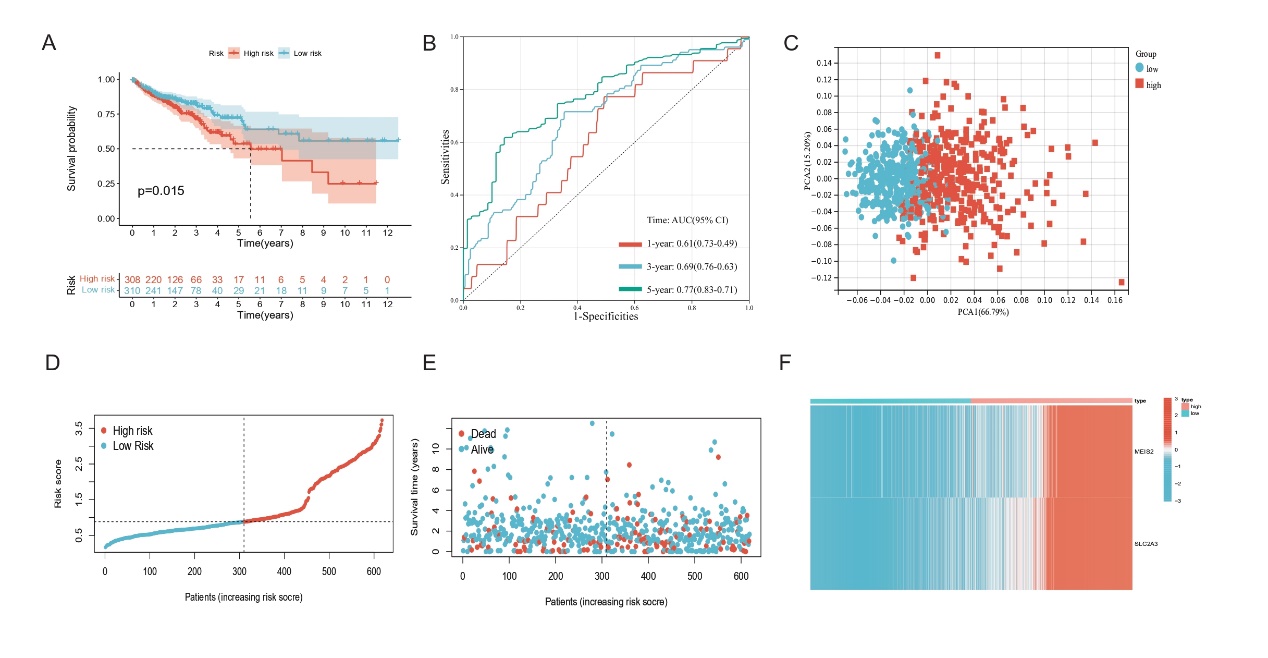


Figure S3 | Validation of EAG_score in test cohort.
